# Association between *Mycobacterium tuberculosis* genotype and diabetes mellitus/hypertension: a molecular study

**DOI:** 10.1186/s12879-022-07344-z

**Published:** 2022-04-24

**Authors:** Shengqiong Guo, Shiguang Lei, Prasit Palittapongarnpim, Edward McNeil, Angkana Chaiprasert, Jinlan Li, Huijuan Chen, Weizheng Ou, Komwit Surachat, Wan Qin, Siyu Zhang, Rujuan Luo, Virasakdi Chongsuvivatwong

**Affiliations:** 1 Guizhou Provincial Center for Disease Control and Prevention, Guiyang, China; 2 Department of Epidemiology, Faculty of Medicine, Prince of Songkla University, Hat Yai, Songkhla Thailand; 3 Guiyang Public Health Clinical Center, Guiyang, China; 4 Department of Microbiology, Faculty of Science, Mahidol University, Bangkok, Thailand; 5 National Science and Technology Development Agency, Pathum Thani, Thailand; 6 Department of Research and Development Affairs, Faculty of Medicine, Siriraj Hospital, Mahidol University, Bangkok, Thailand; 7 Molecular Evolution and Computational Biology Research Unit, Faculty of Science, Prince of Songkla University, Songkhla, Thailand; 8 Division of Computational Science, Faculty of Science, Prince of Songkla University, Songkhla, Thailand; 9 Liupanshui Center for Disease Control and Prevention, Liupanshui, China

**Keywords:** Association, Genotype, Lineage, Drug resistance, Tuberculosis, NCD, MIRU-VNTR, COVID-19

## Abstract

**Background:**

A paucity of studies focused on the genetic association that tuberculosis (TB) patients with non-communicable diseases (NCDs) are more likely to be infected with *Mycobacterium tuberculosis* (MTB) with more potent virulence on anti-TB drug resistance than those without NCDs. The study aimed to document the predominant genotype, determine the association between MTB genotypes and NCD status and drug resistance.

**Methods:**

We conducted a molecular study in 105 TB patients based on a cross-sectional study focused on the comorbid relationship between chronic conditions and TB among 1773 subjects from September 1, 2019 to August 30, 2020 in Guizhou, China. The participants were investigated through face-to-face interviews, followed by NCDs screening. The DNA of MTB isolates was extracted prior to genotyping using 24 loci MIRU-VNTR. The subsequent evaluations were performed by phylogenetic trees, combined with tests of statistical power, Chi-square or Fisher and multivariate logistic regression analysis.

**Results:**

The Beijing family of Lineage 2 (East Asia) was the predominant genotype accounting for 43.8% (46/105), followed by Lineage 4 (Euro-America) strains, including Uganda I (34.3%, 36/105), and the NEW-1 (9.5%, 10/105). The proportion of Beijing strain in patients with and without NCDS was 28.6% (8/28) and 49.4% (38/77), respectively, with a statistical power test value of 24.3%. No significant association was detected between MTB genotype and NCD status. A low clustering rate (2.9%) was identified, consisting of two clusters. The rates of global, mono-, poly- and multi-drug resistance were 16.2% (17/105), 14.3% (15/105), 1.0% (1/105) and 4.8% (5/105), respectively. The drug-resistant rates of rifampicin, isoniazid, and streptomycin, were 6.7% (7/105), 11.4% (12/105) and 5.7% (6/105), respectively. Isoniazid resistance was significantly associated with the Beijing genotype of Lineage 2 (19.6% versus 5.1%).

**Conclusions:**

The Lineage 2 East Asia/Beijing genotype is the dominant genotype of the local MTB with endogenous infection preponderating. Not enough evidence is detected to support the association between the MTB genotype and diabetes/hypertension. Isoniazid resistance is associated with the Lineage 2 East Asia/Beijing strain.

**Supplementary information:**

The online version contains supplementary material available at 10.1186/s12879-022-07344-z.

## Background

The two principal components used to treat tuberculosis (TB) caused by *Mycobacterium tuberculosis* (MTB) are isoniazid and rifampicin. If both of them failed to treat TB, multiple drug resistance TB (MDR-TB) would develop, which would be a disaster not only to the TB individual but also to the community [1]. Currently, there are at least three theories explaining the mechanisms of MTB drug resistance[2], namely the accumulation of gene mutations [3–5], the development of efflux pumps [6–9] and the acceleration of mutations due to the DNA damage repair system against the host cellular defense [6, 7, 10–12]. All of this light of the importance of using genetic markers to identify resistant strains.

Strain genotype information of MTB is required to provide additional evidence of whether a transmission event has occurred. The Beijing strain of MTB is presented as the predominant MTB strain. It plays a vital role in many countries, such as Bangladesh (26.8%) [13], Upper Myanmar (71.4%) [14] and China (81.7%) [15], which holds the second high tuberculosis burden accounted for 8.5% of the case notifications worldwide [16].

The Beijing strain was considered that it might be more virulent, pathogenic, faster-growing, with more histopathological changes and drug resistance tendencies than other strains [17] as well as having a higher mortality rate. This might be due to the unique properties of protein and lipid structures and their interactions with the human immune system [18] particularly when the hosts comorbid with specific chronic conditions. However, there is still a need to populate more evidence.

Diabetes mellitus (DM) and hypertension, as well as their risk factors [19], were reported to play essential roles in the process of TB condition[20]. In China, the proportion of deaths caused by non-communicable diseases (NCDs) increased from 89.82 to 91.41%, with an average annual increase of 0.1% (95%Cl: 0.1-0.2%) [21]. People with DM or hypertension were found to present a higher prevalence among TB patients than those without[22], particularly in those infected by the MTB strains with anti-TB drug resistance mutations [23]. According to some previous studies, the Beijing strains might contain a kind of conserved gene with more expressions by releasing specific cytokines to trigger the pathogenesis of chronic diseases related to host immunities [24, 25].

There is, however, a contrary report suggesting that there is insufficient evidence to conclude that the Beijing strains are more infective or drug-resistant than the non-Beijing strains [15]. The relationship between genotype and TB drug resistance remains elusive.

Therefore, it is necessary to explore the relationship between the genotype of MTB and hosts with specific chronic conditions as well as the drug resistance phenotype, especially during the COVID-19 pandemic. Previous studies have shown that patients with DM/hypertension and TB are twice as likely to be infected with the COVID-19 virus [26], with a prolonged recovery period paralleling more severe complications and sequelae [27] and higher mortality than the general population [28].

Therefore, we hypothesize that the MTB genotypes may differ between TB patients with and without DM and hypertension. We aimed to (1) describe the predominant genotype of MTB based on mycobacterial interspersed repetitive unit-variable number tandem repeat (MIRU-VNTR) sequencing; (2) explore the possible characteristics associated with genotypes of MTB; (3) determine the association between the DM/hypertension status and the genotype of MTB; and (4) test the linkage between MTB genotype and drug susceptibility. Few previous studies have targeted the association between various NCD status and MTB genotypes, making this study the first of its kind.

## Methods

### Study design and sampling process

This cross-sectional study was a part of a study[29, 30] focused on the comorbid relationship between chronic conditions and TB among 801 TB patients retrieved from the TB management system and confirmed through chest X-ray/smear-positive/symptom/signs and 972 related household contacts. The participants were drawn consecutively from 11 counties and districts out of 88 counties, Guizhou from September 1, 2019 to August 30, 2020.

All the participants were face-to-face interviewed at home and underwent body examinations in local hospitals. Later, we contacted TB hospitals and the Centers for Disease Control and Prevention in Guizhou, where the MTB cultures were conducted and the isolates were stored. The essential information of the subjects, including name, gender, age, ID number when available, and household address, were record-linked between the system and the database isolates conserved. Ultimately, 105 TB patients with sputum culture-positive with complete information were record-linked successfully.

### Inclusion and exclusion criteria

#### Inclusion criteria

Newly diagnosed TB index cases aged ≥15 years treated for 0–6 months and notified to the national tuberculosis program system from the research locations.

#### Exclusion criteria

TB patients who were on their retreatment regimen for TB or were pregnant, mentally retarded, or lived alone.

### Relevant definitions

Hypertension: Systolic blood pressure (SBP) ≥ 140 mmHg and/or diastolic blood pressure (DBP) ≥ 90 mmHg or with a history of previously known disease per WHO criteria. Prehypertension: SBP 130 ~ 139 mmHg and/or DBP 85 ~ 89 mmHg [31].

DM: Fasting plasma glucose (FPG) ≥ 126 mg/dl or random plasma glucose (RPG) ≥ 200 mg/dl or with a previous diagnosis of DM. Prediabetes: FBG ≥ 110 mg/ dl but < 126 mg/ dl according to American Diabetes Association (2016) [32].

NCDs: Refers to non-communicable diseases, mainly including DM and/or hypertension in this study. Other NCDs refer to chronic obstructive pulmonary diseases (COPD), heart disease, or dyslipidemia.

Salt intake limit: Over 6 g/day/adult according to the Dietary Guidelines for Chinese Residents (2016) [33].

Oil intake limit: Over 30 g/day/adult according to *the Dietary Guidelines for Chinese Residents (2016)* [33].

Smoking: Smoking in the past 12 months, including both daily and non-daily smoking.

Drinking: Drinking in the past 12 months, including both daily and non-daily drinking.

Regularly serve meat: Serving meat at least one meal per day for three days or above per week.

Newly diagnosed TB cases: Patients with TB and had never been treated with anti-TB drugs or had received anti-TB treatment for less than one month [34].

Cluster: Indistinguishable loci ≥ two of 24 loci MIRU-VNTR strains, or with at least one case with a complete 24 loci MIRU-VNTR profile, and the additional case in the cluster may have one missing locus [35].

### Study flow chart

**Fig. 1:**
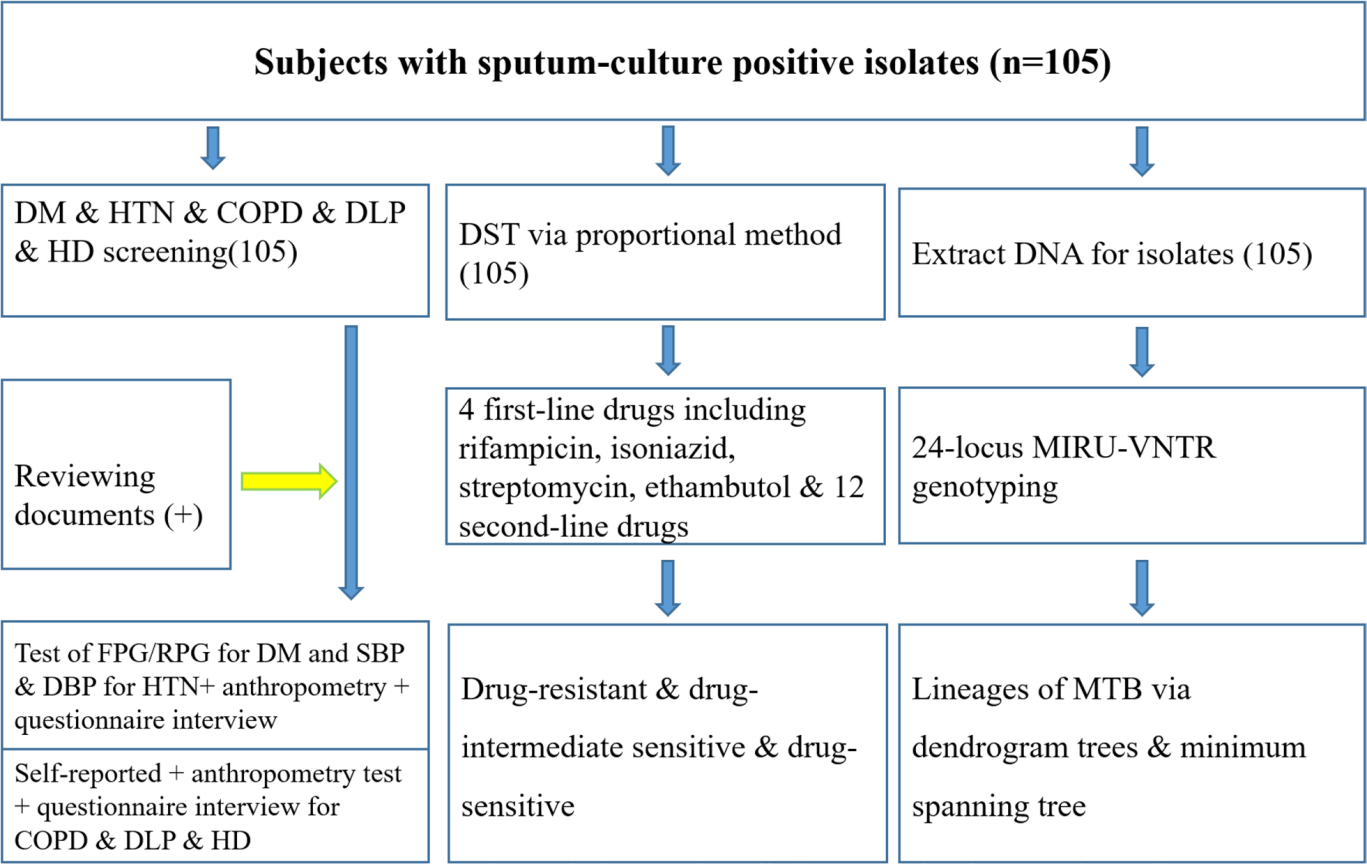
Flow chart of the study on the genetic diversity and drug susceptibility of MTB with and without NCDs. *TB* tuberculosis, *DM* diabetes mellitus, *HTN* hypertension, *COPD* chronic obstructive pulmonary disease, *DLP* dyslipidemia, *HD* heart disease, *FPG* fasting plasma glucose, *RPG* random plasma glucose, *SBP* systolic pressure, *DBP *diastolic pressure, *DST *drug susceptibility test, *MIRU-VNTR *mycobacterial interspersed repetitive unit-variable number tandem repeat

Figure 1 displays the research procedures, including screening for NCDs, 24 loci MIRU-VNTR assay genotyping and 16-drug susceptibility testing (DST) (Fig. 1).

### Sample size

The two independent means formula was used to determine the minimum sample size.

$$n_1={\textstyle\frac{\left(z_{1-\frac\alpha2}+z_{1-\beta}\right)^2\left[\sigma_1^2+{\textstyle\frac{\sigma_2^2}r}\right]}{\triangle^2}}$$

$$r=\frac{n_2}{n_1},\;\triangle=\mu_1-\mu_2$$

where n_1_ is the number of MTB of subjects with NCDs; n_2_ is the number of MTB of participants without NCDs; and *r* is the ratio n_2_/n_1_ = 2.75; *µ*_1_ = 0.3430, the assumed distance from the neighbor MTB lineages of subjects with NCDs; *σ*_2_ = 0.007; *µ*_2_ = 0.3478, the assumed distance from the neighbor MTB lineages of subjects without NCDs; *σ*_1_ = 0.007. The type I error rate (α) = 0.05; and the type II error rate (β) = 0.20.

The formula resulted in n_1_ = 23, n_2_ = 64. Considering a 10% non-response rate and a 10% of samples being broken or contaminated, ultimately, 104 subjects were planned to be recruited.

### Screening for NCDs

During the monthly visits of TB patients to the hospital to obtain their medication, TB medical staff approached them, introduced themselves and explained the study objectives. Those willing to participate were asked to provide written informed consent to investigate their NCDs and sputum collection and testing, and then an appointment was made with them for a home visit.

After completing an interview with a structured questionnaire that included socio-demographic, behavioral and clinical characteristics, participants were transferred to the related clinics for the appropriate laboratory tests. DM screening was performed through FPG/RPG; hypertension was screened through SBP & DBP, whereas COPD, dyslipidemia and heart disease were screened through self-reporting due to practical restrictions. The medical documentation reporting to the above NCDs was considered as screened positive. All newly diagnosed cases with confirmed NCD were transferred to the relevant hospitals for further treatment.

### Molecular genotyping and analysis for MTB

Cultures of MTB were performed at the Reference Mycobacteriology Laboratory following the standard criteria [36]. Cultures were grown on a Löwenstein-Jensen (LJ) medium for 6–8 weeks or on MGIT culture for two weeks. Mycobacterial Deoxyribonucleic acid (DNA) extraction was performed among the MTB fresh sub-cultures as described elsewhere[37].

Subsequently, DNA was sent to the gene-sequencing company (Beijing Tianyi Huiyuan Life Science & Technology Inc.) for MIRU-VNTR assay using the numerical code MTBCC15-9 based on 15 conventional discriminatory loci and nine auxiliary highly polymorphic loci for Beijing type of MTB [38]. Firstly, Polymerase chain reaction (PCR) amplification was carried out with H37Rv standard strain (American GenBank ATCC 27,294, preserved by Guizhou Provincial Center for Disease Control and Prevention) as the positive control and H_2_O as the negative control. Then the Applied Biosystems 3730XL DNA Sequencer (AMI of USA) was applied for capillary electrophoresis. The DNA information was edited and imported into the system. GeneMapper v4.0 software was employed to analyze the *fsa* files obtained by the sequencer. Table 1 shows the primer sequence of the MIRU-VNTR loci in this study (Table 1).

**Table 1:** Primer sequence of 24 MIRU-VNTR loci in this study

Locus	Primer	Marker	PCR primer pairs (5’-3’)	Flank size	Repeat unit length (bp)
MIRU02	MIRU02-F	FAM	CAGGTGCCCTATCTGCTGACG	189	47
MIRU04	MIRU04-F	HEX	GTCAAACAGGTCACAACGAGAGGAA	105	77
MIRU10	MIRU10-F	TAMRA	ACCGTCTTATCGGACTGCACTATCAA	219	53
MIRU16	MIRU16-F	FAM	CGGGTCCAGTCCAACTACCTCAAT	367	52
MIRU20	MIRU20-F	HEX	CCCCTTCGAGTTAGTATCGTCGGTT	220	72
MIRU23	MIRU23-F	TAMRA	CGAATTCTTCGGTGGTCTCGAGT	79	52
MIRU24	MIRU24-F	FAM	GAAGGCTATCCGTCGATCGGTT	312	53
MIRU26	MIRU26-F	HEX	GCGGATAGGTCTACCGTCGAAATC	243	48
MIRU27	MIRU27-F	TAMRA	TCTGCTTGCCAGTAAGAGCCA	269	52
MIRU31	MIRU31-F	FAM	CGTCGAAGAGAGCCTCATCAATCAT	108	52
MIRU39	MIRU39-F	HEX	CGGTCAAGTTCAGCACCTTCTACATC	191	47
MIRU40	MIRU40-F	TAMRA	GATTCCAACAAGACGCAGATCAAGA	226	50
ETRA	ETRA-F	FAM	AAATCGGTCCCATCACCTTCTTAT	196	75
ETRB	ETRB-F	FAM	ATGGCCACCCGATACCGCTTCAGT	347	57
ETRC	ETRC-F	HEX	CGAGAGTGGCAGTGGCGGTTATCT	102	58
Mtub04	Mtub04-F	HEX	GTCCAGGTTGCAAGAGATGG	117	51
Mtub21	Mtub21-F	TAMRA	AGATCCCAGTTGTCGTCGTC	150	57
Mtub29	Mtub29-F	HEX	GCCAGCCGCCGTGCATAAACCT	392	57
Mtub30	Mtub30-F	FAM	CTTGAAGCCCCGGTCTCATCTGT	275	44
Mtub34	Mtub34-F	HEX	GGTGCGCACCTGCTCCAGATAA	323	54
Mtub39	Mtub39-F	TAMRA	CGGTGGAGGCGATGAACGTCTTC	284	52
QUB11b	QUB11b-F	FAM	CGTAAGGGGGATGCGGGAAATAGG	67	69
QUB26	QUB26-F	TAMRA	AACGCTCAGCTGTCGGAT	153	111
QUB4156	QUB4156-F	TAMRA	TGACCACGGATTGCTCTAGT	563	59

*24 loci MIRU-VNTR* Mycobacterial interspersed repetitive unit-variable number tandem repeat (MIRU-VNTR) sequencing. Sequencing order: MIRU02 - Mtub04 – ETRC - MIRU04 - MIRU40 - MIRU10 - MIRU16 -Mtub21 - MIRU20 - QUB11b – ETRA - Mtub29 - Mtub30 – ETRB - MIRU23 - MIRU24 - MIRU26 - MIRU27 - Mtub34 - MIRU31 - Mtub39 - QUB26 - QUB4156 - MIRU39

### Drug susceptibility test

The drug susceptibility test of the MTB strains targeted to the four first-line and other 12 anti-TB drugs was performed using the proportional laboratory method following the conventional recommendations of WHO [36].

The drugs and related concentrations in media were applied as below. Isoniazid (INH) 0.4 µg/ml, rifampicin (RFP) 4 µg/ml, ethambutol (EMB) 5 µg/ml, streptomycin (SM) 8 µg/ml, rifapentine (Rft) 2 µg/ml, levofloxacin (Lfx) 8 µg/ml, amikacin (Amk) 4, protionamide (Pto) 40 µg/ml, diphasic (Dip) 2 µg/ml, moxifloxacin (Mfx) 2 µg/ml, capreomycin (CPM) 10 µg/ml, paza-aminosalicylate (PAS) 8 µg/ml, clarithromycin (Clr) 16 µg/ml, Rifabutin (Rfb) 3 µg/ml, kanamycin (KM) 10 µg/ml and clofazimine (Cfz) 8 µg/ml.

Pyrazinamide (PZA) was not included due to its unstable attribute to this method named liquid microporous plate techniques for drug susceptibility.

The MDR-TB strain was defined as resistant to at least both INH and RFP, identified to be resistant to the specific drug when the growth rate was > 1.0% compared to the control group without any drugs [39]. The MDR-TB strains were defined as resistant to at least both INH and RFP. The products related were purchased from Zhuhai Encode (Zhuhai Encode Medical Engineering Co., Ltd).

### Statistical analysis

Data obtained from the questionnaire and record review were entered into EpiData version 3.1 (http://www.epidata.dk/). R version 3.6.3 (https://cran.r-project.org/) was used for all statistical analyses. Quantitative variables, such as age, monthly income, FPG, RPG, SBP and DBP, were cut from continuous exposure variables to create the new categorical variables, which have significant consequences for the later analyses. Categorical variables were analyzed with the Chi-square test or Fisher test.

To further examine the relationship between NCDs status and MTB genotypes, the genetic pattern was classified into Beijing and non-Beijing groups to perform univariate analysis and multiple logistic regression tests. The statistical power test for two proportions was employed to check the proportions of genotypes and the prevalence of NCDs.

Isolates with more than two MIRU-VNTR loci that failed in genotyping were excluded from the analysis. Analysis of MIRU-VNTR *plus* (http://www.miru-vntrplus.org/) was employed for the analysis of the MIRU-VNTR profile by generating a map of sub-lineages, clonal complexes and identification for clusters of MTB [40]. The analysis was conducted with a relaxing average cut-off value of 0.34 by the similarity matching for the numbers of repeated units of DNA. Later, the genetic information was posteriorly confirmed by the unweighted pair group method with arithmetic means (UPGMA) tree-based analysis, displaying with dendrogram and radial trees [40].

The Hunter-Gaston discriminatory index (HGDI) was used to detect the discriminatory power of each locus as follows [41]:

$$HGDI=1-\left[\frac1{N(N-1)}{\textstyle\sum_{n=1}^s}\;n_j\;(n_j-1)\right]$$

where N is the total number of strains in the typing scheme, *s* is the number of distinct patterns discriminated by MIRU-VNTR, and *n*_*j*_ is the number of strains belonging to the *j*^th^ pattern.

The clustering rate is denoted as a percentage computed with the following formula [42].

$$Clustering\;rate=(N_c-C)/N$$

where N, the total number of the isolates, is 105; C, the number of the clusters, is 2; and N_c_, the total number of clustered strains, is 5 [43].

### Guidelines and regulations statement and consent to participate

We confirm that all the methods in this article were carried out in accordance with the relevant human guidelines and regulations. Before this study was conducted, written informed consent was obtained from each participant included. For participants under the age of 18, the information sheets were sent to their parents or legal guardians. All investigations relating to them can only be initiated with the written permission of informed consent of their parents or legal guardians.

## Results

Of the 801 TB patients, there were 243 participants with sputum-cultures positive identified and 170 of them were matched with isolates re-cultured successfully and underwent drug resistance test simultaneously. DNA was extracted from 170 isolates and was sent to Tianyi Gene Sequencing Company for MIRU-VNTR analysis. Eventually, the information of the MIRU-VNTR profile of 105 strains was available for data analysis. The average age of the 105 participants was 45.5 ± 19.8 years, with 64.8% (68/105) male.

### Genetic diversity and HGDI discriminatory power

Table 2 displays the allelic diversity of each locus evaluated by the 24 loci MIRU-VNTR. Locus QUB11b was identified as the most distinctive (HGDI = 0.8040). The loci with the least discriminatory power were MIRU 02 and MIRU 24 (HGDI = 0.0000). The global HGDI discriminatory power was 0.9747, indicating that our strains are relatively distinguishable [42] (Table 2).

**Table 2:** Discriminatory power of 24-locus MIRU-VNTR Loci

Locus/Alias	Number of strains at different Loci										HGI
	0	1	2	3	4	5	6	7	8	9	
QUB11b	0	6	7	19	14	13	37	8	1	0	0.8040
QUB26-R	0	0	0	0	11	7	29	19	25	1	0.7723
Mtub21	32	6	17	32	15	1	0	0	0	0	0.7624
MIRU26	7	8	1	1	8	21	14	45	0	0	0.7495
MIRU40	0	4	27	64	9	1	0	0	0	0	0.5588
MIRU31	0	2	4	30	6	63	0	0	0	0	0.5586
Mtub04	0	0	0	26	15	63	0	0	0	0	0.5551
MIRU39	0	3	35	63	4	0	0	0	0	0	0.5317
MIRU10	0	2	30	67	2	4	0	0	0	0	0.5139
ETRA	0	0	6	22	71	5	1	0	0	0	0.4980
Mtub39	0	15	71	14	3	0	0	0	0	0	0.4891
QUB4156	0	0	66	7	24	0	0	0	0	0	0.4755
Mtub30	0	0	30	1	0	72	1	1	0	0	0.4522
MIRU16	0	3	11	84	7	0	0	0	0	0	0.3471
MIRU04	2	5	87	1	10	0	0	0	0	0	0.3046
MIRU27	0	2	15	87	0	0	0	0	0	0	0.2817
ETRB	0	15	89	1	0	0	0	0	0	0	0.2636
Mtub34	1	0	9	92	3	0	0	0	0	0	0.2262
MIRU23	0	0	0	0	2	102	1	0	0	0	0.0564
ETRC	0	0	0	0	3	102	0	0	0	0	0.0560
MIRU20	0	1	103	1	0	0	0	0	0	0	0.0379
Mtub29	0	103	2	0	0	0	0	0	0	0	0.0377
MIRU02	0	0	105	0	0	0	0	0	0	0	0.0000
MIRU24	0	90	0	0	0	0	0	0	0	0	0.0000

*24 loci MIRU-VNTR* Mycobacterial interspersed repetitive unit-variable number tandem repeat (MIRU-VNTR) sequencing. Each-digit represents the number of repeats at a particular locus according to the following order of the loci: MIRU02 - Mtub04 – ETRC - MIRU04 - MIRU40 - MIRU10 - MIRU16 -Mtub21 - MIRU20 - QUB11b – ETRA - Mtub29 - Mtub30 – ETRB - MIRU23 - MIRU24 - MIRU26 - MIRU27 - Mtub34 - MIRU31 - Mtub39 - QUB26 - QUB4156 - MIRU39. *HGDI* Hunter-Gaston discriminatory index

### Profile of genotypes and clusters of MTB

**Fig. 2:**
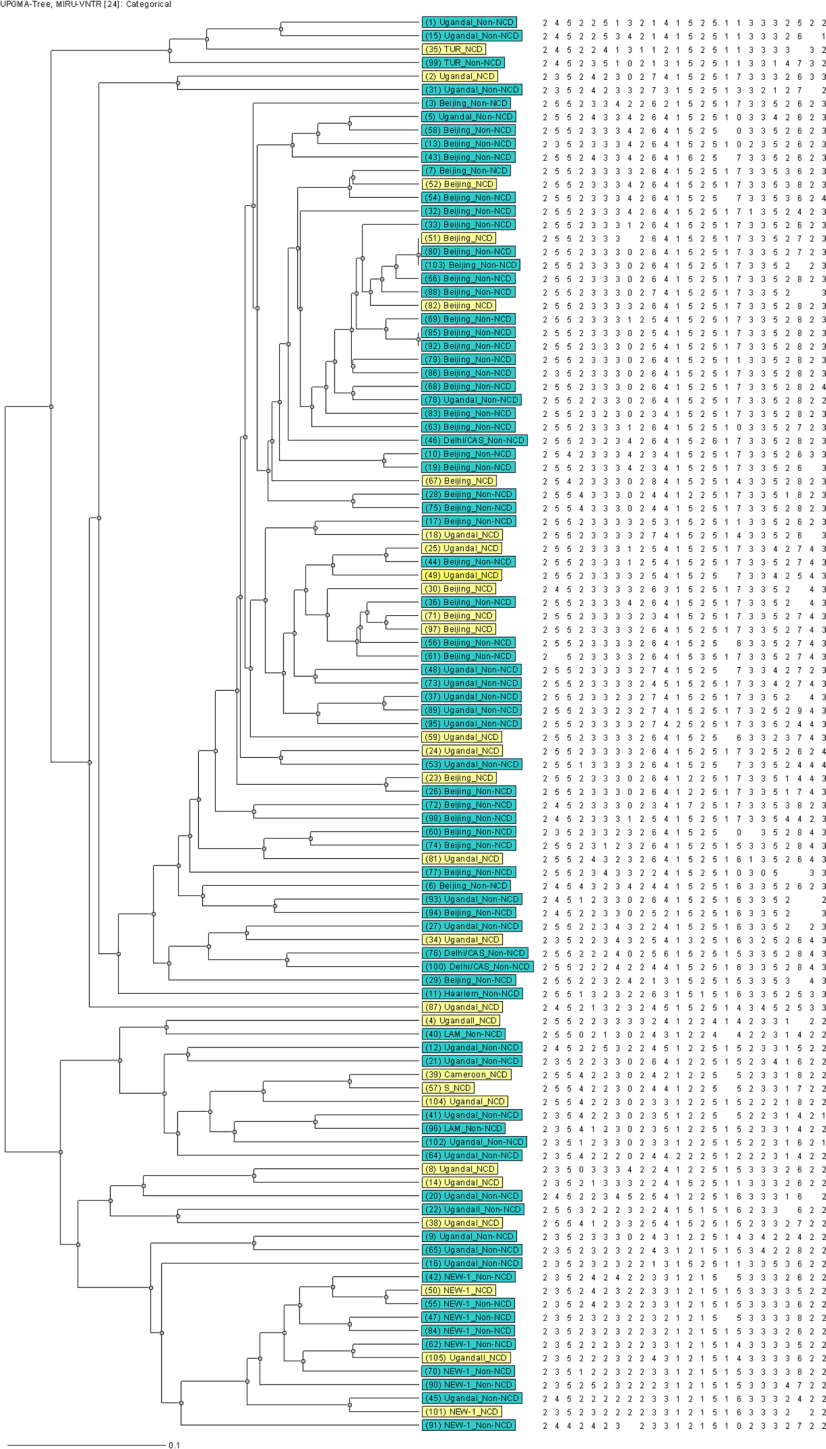
UPGMA dendrogram tree of MTB with NCD status. The light-yellow color stands for subjects with NCD(s), whereas the green represents participants without NCDs. *MTB* *Mycobacterium tuberculosis*, *NCDs* non-communicable chronic diseases, refers to DM, HTN, dyslipidemia, heart disease and chronic obstructive pulmonary disease

**Fig. 3:**
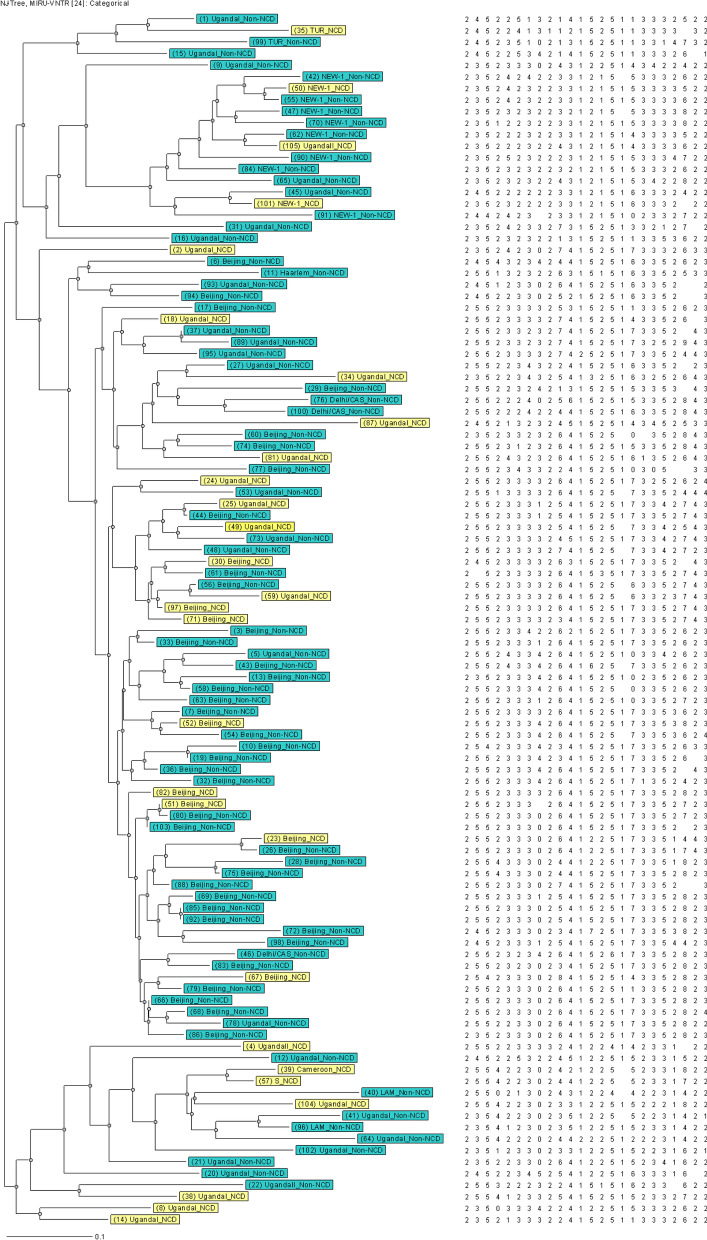
Neighbor-joining dendrogram tree of MTB with NCD status. The light-yellow color stands for subjects with NCD(s), whereas the green represents participants without NCDs. *MTB* *Mycobacterium tuberculosis*, *NCDs* non-communicable chronic diseases, refers to DM, HTN, dyslipidemia, heart disease and chronic obstructive pulmonary disease

**Fig. 4:**
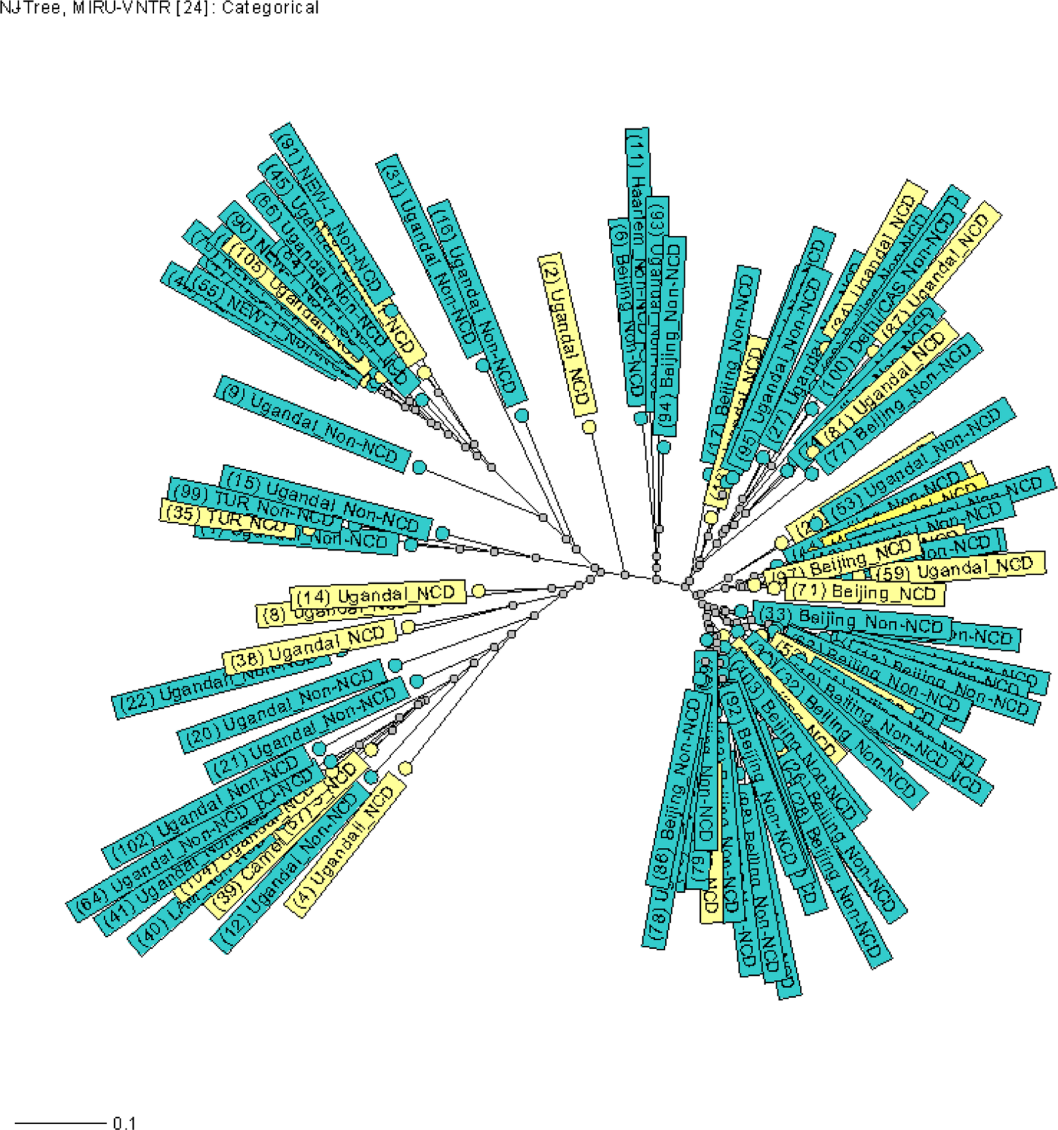
Neighbor-joining radial tree of MTB with NCD status. The light-yellow color stands for subjects with NCD(s), whereas the green represents participants without NCDs. *MTB* *Mycobacterium tuberculosis*, *NCDs* non-communicable chronic diseases, refers to DM, HTN, dyslipidemia, heart disease and chronic obstructive pulmonary disease

The genotypes of MTB are displayed through UPGMA (Fig. 2) and Neighbor-joining (Fig. 3) dendrogram trees and radial tree (Fig. 4) as well stratified by NCD status. The Beijing family of Lineage 2 (East Asia) was the predominant genotype at 43.8% (46/105), followed by the Lineage 4 (Euro-America) strains, including Uganda I (34.3%, 36/105), and the NEW-1 (9.5%, 10/105).

Lineage 4, including Uganda II (2.9%, 3/105), Latin American-Mediterranean (LAM, 1.9%, 2/105), TUR (1.9%, 2/105), Cameroon (1.0%, 1/105), Haarlem (1.0%, 1/105) and the S (1.0%, 1/105) strains were identified. Delhi/Central Asian, CAS (2.9%, 3/105), belonging to Lineage 3 was also detected through the best matching of similarity. The ones with NCDs scattering throughout the tree suggests no significant association between these MTB genetic patterns and NCD status.

There were two distinct clusters evident with a clustering rate of 2.9%. The first consisted of three strains (No. 51, 80 and 103), whereas the second had two members (No. 85 and 92). Both are from the Beijing family of Lineage 2 (East Asia) (Figs. 2, 3 and 4).

### Minimum spanning tree/multi-dimensional scaling map by NCD status

**Fig. 5:**
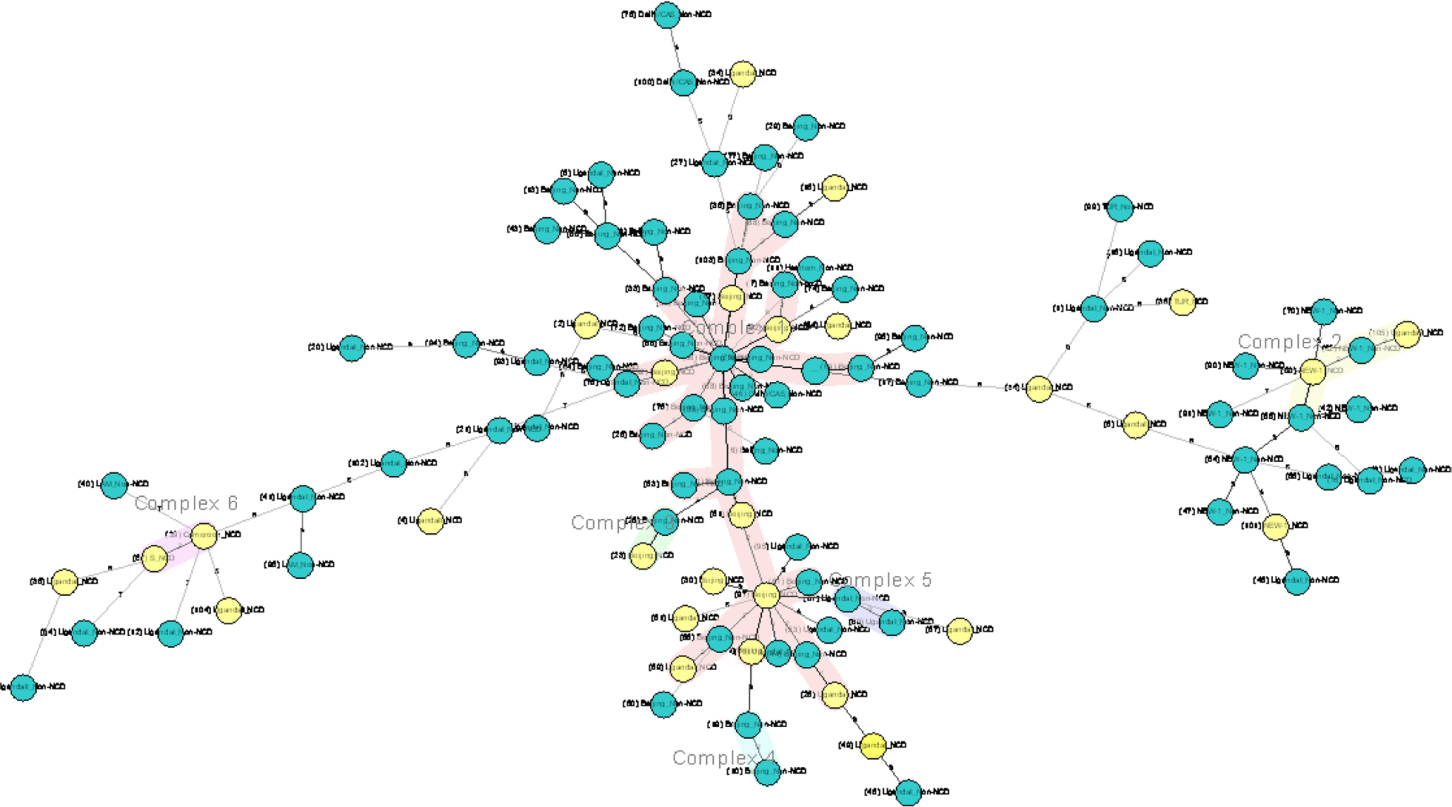
Minimum spanning tree of MTB with NCD status. The light-yellow color stands for subjects with NCD(s), whereas the green represents participants without NCDs. *MTB* *Mycobacterium tuberculosis*, *NCDs* non-communicable chronic diseases, refers to DM, HTN, dyslipidemia, heart disease and chronic obstructive pulmonary disease.

Figure 5 shows the minimum spanning tree of the genotypes created using Kruskal’s algorithm and a force-directed graph layout [40, 44] with NCD status. There were six clonal complexes (CCs), with the proportions of DM, hypertension and other NCDs in CC1 being 25.0% (n = 7), 10.7% (n = 3), and 10.7% (n = 3), respectively, and that in singletons was 16.9% (n = 11), 9.2% (n = 6) and 7.7% (n = 5), respectively. The size of strains with NCDs distributed in CC2-CC5 was 0–1. No specific association between clonal complex and NCD status is evidenced (Fig. 5).

**Fig. 6:**
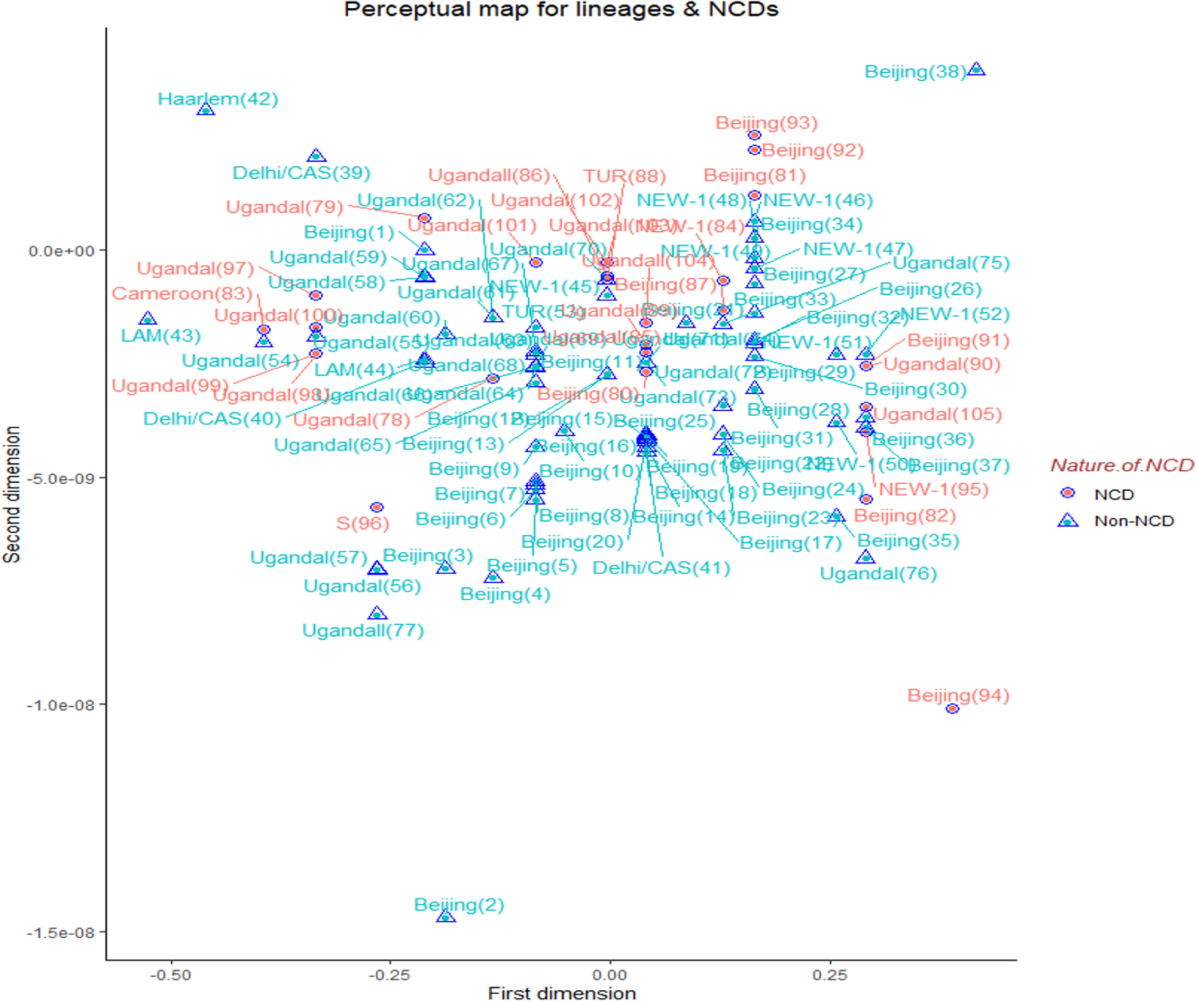
Multi-dimensional scaling perceptual map of MTB genotypes with NCD status. *MTB* *Mycobacterium tuberculosis*, *NCDs* non-communicable chronic diseases, refers to DM, HTN, dyslipidemia, heart disease and chronic obstructive pulmonary disease.

Figure 6 displays a multi-dimensional scaling map of MTB Genotypes by NCD Status. The scattered signs suggest no distinguishable location difference between the subjects with NCDs and those without NCDs (Fig. 6).

### Genotypes profile by different characteristics

To further examine the relationship between NCDs status and MTB genotypes, we classified the genetic patterns of MTB into Beijing and non-Beijing groups to perform the analyses of statistical power, univariate and multiple logistic regression.

The percentages of Beijing strain among subjects with and without NCDs were 28.6% (8/28) and 49.4% (38/77), respectively. The statistical power test for the two groups revealed that the statistical power value was 24.3% (*P* value = 0.058, odds ratio [OR] = 0.41, 95%CI = 0.16, 1.04), indicating a weak statistical test power. The OR estimate was 0.41 (CI 95% 0.16, 1.04). The wide confidence interval indicates that there is not enough evidence to draw conclusions about the association between the genotype of MTB and NCD status with the weak statistical test power.

Table 3 summarizes the relationships between the Beijing genotype and specific NCDs as well as gender and age brackets. In the univariate analysis, no significant association was found among the most variables related to NCD status contributing to Beijing genotypes of MTB. The males were more likely to be infected by the Beijing family compared to females, who were mainly infected by Uganda I genotype (Table 3).

**Table 3:** Lineages of MTB among patients by NCDs and other factors (n, %)

Variables		Total	Beijing	Non-Beijing			OR	*P* value
				Uganda I	NEW-1	Other Lineages		
Total		105	46	36	10	13		
DM ^*^	No	87	36 (41.4)	30 (34.5)	10 (11.5)	11 (12.6)	Ref.	0.482
	Yes	18	10 (55.6)	6 (33.3)	0 (0.0)	2 (11.1)	1.030	
HTN	No	93	40 (43.0)	31 (33.3)	9 (9.7)	13 (14.0)	Ref.	0.649
	Yes	12	6 (50.0)	5 (41.7)	1 (8.3)	0 (0.0)	1.330	
Other NCDs	No	96	41 (42.7)	34 (35.4)	10 (10.4)	11 (11.5)	Ref.	0.540
	Yes	9	5 (55.6)	2 (22.2)	0 (0.0)	2 (22.2)	1.030	
Gender	Female	37	12 (32.4)	17 (45.9)	7 (18.9)	1 (2.7)	Ref.	0.003
	Male	68	34 (50.0)	19 (27.9)	3 (4.4)	12 (17.6)	1.040	
Age (year-old)	15~34	40	17 (42.5)	13 (32.5)	6 (15.0)	4 (10.0)	Ref.	0.285
	35~59	35	12 (34.3)	14 (40.0)	2 (5.7)	7 (20.0)	0.520	
	60~100	29	17 (58.6)	9 (31.0)	1 (3.4)	2 (6.9)	0.730	

*Other lineages* Uganda II, Delhi/CAS LAM, TUR, Cameroon, Haarlem, and S, *OR* Odds ratio, *DM* Diabetes, *HTN* Hypertension, *COPD* Chronic Obstructive Pulmonary Disease, *NCDs* non-communicable chronic diseases, refers to DM, HTN, dyslipidemia, heart disease and COPD, *Other NCDs* dyslipidemia, heart disease and COPD

**Fig. 7:**
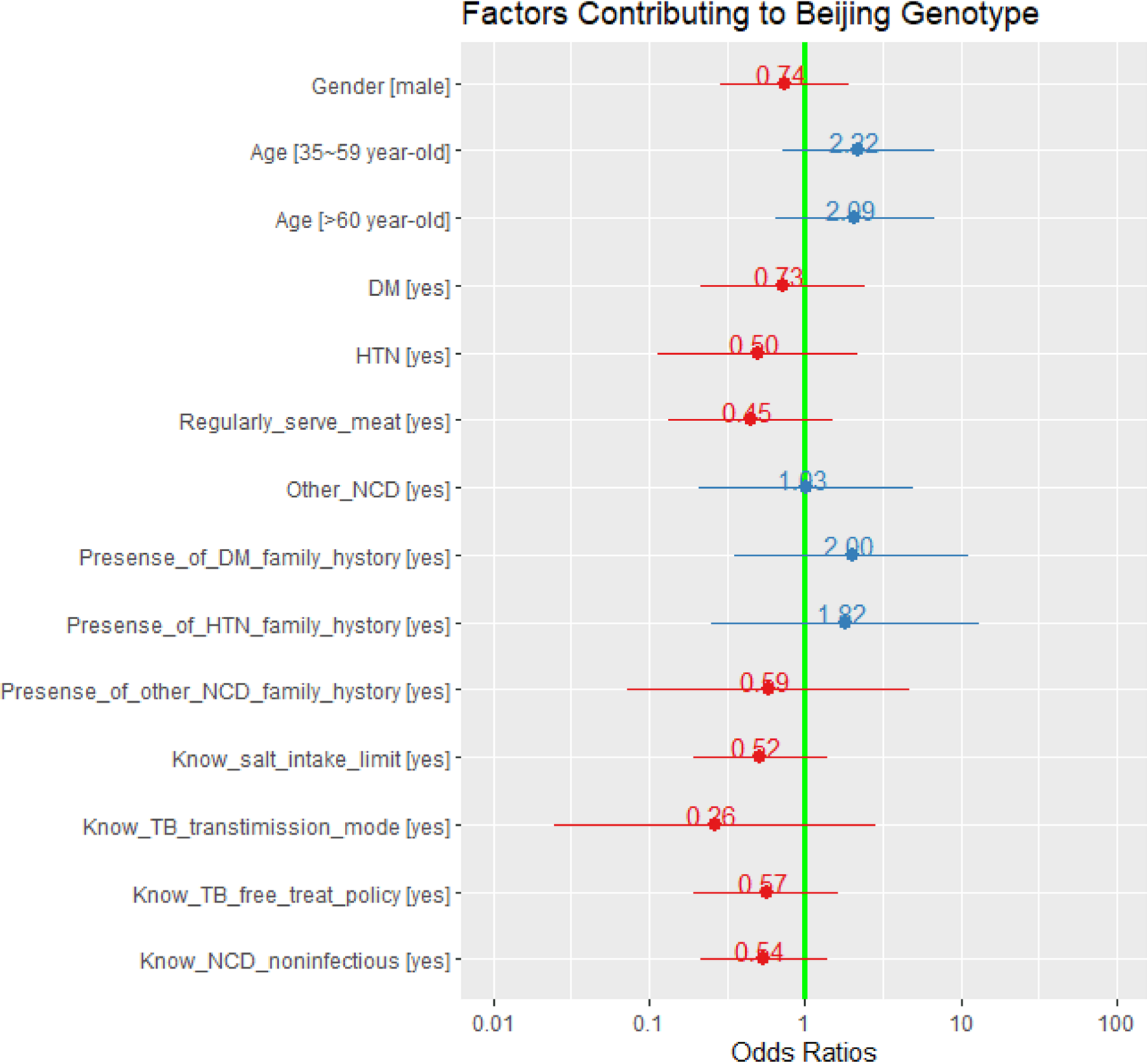
Odds ratios of multivariate logistic regression analysis. All *P* values > 0.05. *DM* Diabetes, *HTN* Hypertension. *NCDs* non-communicable chronic disease, refer to DM, HTN, dyslipidemia, heart disease and chronic obstructive pulmonary disease. *Other NCDs* dyslipidemia, heart disease and chronic obstructive pulmonary disease. Salt intake limit: Over 6 grams/day/adult according to the Dietary Guidelines for Chinese Residents (2016). Regularly serve meat: Here relates to serving meat at least one meal per day for three days or above per week.

Based on the Beijing group as the dependent variable, gender, age brackets and the variables related to NCD as well as those with a *P* value less than 0.2 from the univariate analysis (Additional file 1: Table S1) as the independent variables were included to carry out the multiple logistic regression analysis. There was no association between Beijing genotype and NCD status or any of the socio-demographic and behavioral characteristics (Fig. 7).

### Association between drug resistance and genotypes of MTB

Table 4 presents the association of different types of drug resistance and MTB genotypes. The global, mono-, poly- and multi-drug resistance rates were 16.2% (17/105), 14.3% (15/105), 1.0% (1/105) and 4.8% (5/105). The drug-resistant rates of rifampicin, isoniazid and streptomycin, were 6.7% (7/105), 11.4% (12/105) and 5.7% (6/105), respectively. No resistance to ethambutol was detected in the present study (Additional file 2).

**Table 4:** Association between different types of drug resistance and lineages (n)

Drug-resistant		Beijing	Non-Beijing			*P* value
			Uganda I	NEW-1	Other Lineages	
Total		46	36	10	13	
Any DR	No	35	31	10	12	0.103
	Yes	11	5	0	1	
Mono-DR	No	36	32	10	12	0.100
	Yes	10	d4	0	1	
Poly-DR	No	45	36	10	13	0.438
	Yes	1	0	0	0	
MDR	No	42	35	10	13	0.166
	Yes	4	1	0	0	
Rifampicin	No	41	34	10	13	0.236
	Yes	5	2	0	0	
Isoniazid	No	37	34	10	12	0.045
	Yes	9	2	0	1	
Streptomycin	No	42	34	10	13	0.401
	Yes	4	2	0	0	

*Other lineages* Uganda II, Delhi/CAS LAM, TUR, Cameroon, Haarlem, and S, *DR* Drug resistance, *Mono- DR* Drug resistance to only one first-line anti-TB drugs, *Poly- DR* Drug resistance to more than one first-line anti-TB drugs but not including both isoniazid and rifampicin resistance simultaneously, *MDR* Drug resistance at least to rifampicin and isoniazid simultaneously

The isoniazid resistance was significantly associated with the Beijing genotype of Lineage 2 [19.6% (9/46) versus 5.1% (3/59)]. For other types of drug resistance, there was no significant association between the types of drug resistance and the genotypes of MTB (Table 4).

## Discussion

The Beijing family of Lineage 2 (East Asia) was the predominant genotype, followed by the Lineage 4 (Euro-America) strains of MTB with endogenous infection dominating. The percentage of Beijing strain among subjects with NCDs was lower than that of those without NCDs. No significant association between NCD status and MTB genotype was found. The isoniazid resistance was associated with the Beijing genotype (Additional file 3).

The Beijing genotype of Lineage 2 (East Asia) plays the dominant role in the current study. However, the proportion (43.8%) of Beijing genotype is lower than that found in other provinces or municipalities of China, such as Guangxi (53.2%) [45], Xinjiang (71.2%) [46] and Beijing Municipality (81.0%) [37]. The Beijing genotype is believed that it might confer a type of gene with more expressions, interacting with the host immune system harboring a variant of the Toll-interleukin 2 receptor (TLR2)[24, 25, 47], known to trigger a cytokine cascade upon recognition of MTB, increased TB susceptibility only in patients infected with a Beijing strain, releasing the immunologic substances, such as chemokine 10 (CK10), tumor necrosis factor α (TNF-α) [48], interferon γ (IFN-γ) and interleukin 17 (IL-17) [49]. These cytokines are the effector molecules, adjusting the expression of corresponding genes by activating nuclear transcription factors, thereby regulating the apoptosis of pancreatic β-cells and triggering DM [24, 50, 51].

A previous study suggested that TB-DM and TB-prediabetes patients were more likely to be infected by the Beijing and Haarlem strains [18]. Except for Asian immigration increasing since the last century, a hypothesis might be assumed that there was an existence of mechanisms that allowed the transmission of MTB lineages among affected patients with the same comorbidity or that these patients could be more susceptible to exogenous infection from other patients. It was also reported the Beijing lineage might repress some miRNA expressions, which might play a pivotal role and reflect their virulent characteristics in altering the host response, such as up-regulating those patients with elevated HbA1c and other reactions [50–54]. Factors common to the other NCDs, including oxidative stress, increased interstitial, sodium, cytokine production, and inflammasome activation, promote immune activation in hypertension, however, none of these above hypotheses could be confirmed by our data.

Our study manifests a 24.3% chance of finding a significant difference given that the Beijing strain has an odds ratio of 0.41 to be associated with NCD status, suggesting that the lack of association is inconclusive. This might be interpreted to some extent by the low clustering rate found in our study. Clustering lineages share common attributes, typically proximity according to distance or similarity measures [42]. The clustering rate indicates higher discriminatory power with lower percentages and with less possibility of recent transmission in a local population. Our clustering rate was only 2.9%, meaning that few subjects in our data suffered from the current transmission delivered by the identical lineages of MTB with the same genetic characteristics among the population [55]. Conversely, it might be the consequence of the situation that the NCDs impaired the host’s immune system so that the MTB inside the host bodies previously infected has been reactivated [23]. According to a prospective study, 21 of 26 (80.8%) of the second episode among TB patients with DM were caused by bacteria with the same genotype of MTB indicating endogenous reactivation of MTB, while 5 of 26 instances (19.23%) re-infected with a different strain suggesting recent transmission[56].

What is noteworthy is that the Uganda I genotype ranked the second highest with 34.3% among the patients harboring this kind of strains. According to another study, lineage L4.6/Uganda resulted in more severe TB disease when found together with an ancestral allele in SLC11A1 of the human host [57]. In this study, males were more likely to be infected by the Beijing type of Lineage 2 (East-Asian) than females, who were more likely to be infected by Uganda I of Lineage 4. This is slightly different from the study in Botswana [58], in which gender was positively associated with drug resistance rather than the types of lineage.

Our study showed that Beijing strain was positively correlated with this phenotype, isoniazid resistance, reported to be mainly related to the *katG* gene mutations[59]. This is similar to the findings of studies in a whole-genome sequencing based study in China[60], of which 1024 MDR strains were identified from 2019 strains of *Mycobacterium tuberculosis*. The main mutation types of common drug-resistance related genes were *katG* S315T (73.2%, isoniazid), *rpoB* S450L(63.1%, rifampicin), *rpsL* K43R(70.0%, streptomycin), *embB* M306V(37.4%, Ethambutol, *pncA*_promoter T (-11) C (7.9%, pyrazinamide), *gyrA* A90V (32.3%, fluoroquinolones), *RRS* A1401G(67.7%, second-line injection drug), *fabGl*_promoter C (-15) T (7.0%, ethionamide). Similar findings is available in a study of Iran [61]. In another study, the effect of mutations on the transmission of isoniazid-resistant strains was comparable to the impact of other clinical determinants of transmission [62], such as the selection pressure from inappropriate TB therapy [63].

The present study results provide an insight into the epidemiological and molecular characteristics of patients with MTB comorbid with respective NCDs, which will lay a preliminary foundation for further interdisciplinary research on TB and chronic non-communicable diseases, especially during the COVID-19 pandemic. Previous studies have shown that patients with NCDs and TB are twice as likely to be infected with the COVID-19 virus, with a prolonged recovery period paralleling more severe complications and sequelae and higher mortality than the general population. Similarly, the COVID-19 usually leads to more severe events, such as intensive care unit admission, mechanical intubation, and mortality among people who are comorbid with NCDs/TB than those without the diseases [64].

### Limitations

There are some limitations in the present study. The prevalence of other NCDs, including COPD, heart disease and dyslipidemia, was obtained through self-reported, meaning some biases might exist. The proportion of non-NCD subjects in the study was smaller than what had been estimated during the sample size calculation, which could be a cause of under power in our hypothesis testing. Moreover, the cross-sectional nature of this study could not specify the direction of causation. Prudence should be observed when the results of this study are generalized.

## Conclusions

The Beijing family of Lineage 2 (East Asia) is the dominant genotype circulating MTB with endogenous infection ruling. Not enough evidence is detected to support the association between the MTB genotype and diabetes/hypertension. Isoniazid resistance is associated with the Beijing genotype of Lineage 2 (East Asia).

## Supplementary Information

**Additional file 1. Table S1.** Univariate analysis of factors for Beijing and non-Beijing genotype of Mycobacterium tuberculosis [Beijing vs non-Beijing, n (%)] *.

**Additional file 2.** **Data S1.** Best matching database-strain lineage of similarity.

**Additional file 3.** **Data S2.** Information of 24 loci MIRU-VNTR.

## Data Availability

The data that support the findings of this study are included in the article and within the Supplementary Information.

## Abbreviations

TB: Tuberculosis
MTB: *Mycobacteria tuberculosis*
NCDs: Non-communicable diseases
DM: D iabetes mellitus
HTN: Hypertension
COPD: Chronic obstructive pulmonary disease
DLP: Dyslipidemia
HD: Heart disease
CRD: Chronic Renal Disease
NCDs Family History: Presence of family with a history of DM, HTN, COPD, DLP, HD, Cancer, CRD
Other NCDs: COPD, DLP, HD
FPG: Fasting plasma glucose
RPG: Random plasma glucose
SBP: Systolic pressure
DBP: Diastolic pressure
DST: D rug susceptibility test
MIRU-VNTR: Mycobacterial interspersed repetitive unit-variable number tandem repeat
HGDI: Hunter-Gaston discriminatory index
Other lineages: Uganda II, Delhi/CAS LAM, TUR, Cameroon, Haarlem, and S
