# Supplementary material for: Association between Mycobacterium tuberculosis genotype and diabetes mellitus/hypertension: a molecular study
Source: BMC Infect Dis. 2022 Apr 24;22:401. doi: 10.1186/s12879-022-07344-z (PMC9035274; doi:10.1186/s12879-022-07344-z)
Supplement: Supplementary file 1 — Additional file 1. Table S1. Univariate analysis of factors for Beijing and non-Beijing genotype of Mycobacterium tuberculosis [Beijing vs non-Beijing, n (%)] *. [file 12879_2022_7344_MOESM1_ESM.pdf]

Table S1. Univariate analysis of factors for Beijing and non-Beijing genotype of *Mycobacterium tuberculosis* [Beijing vs non-Beijing, n (%)] *

Variables		Beijing	Non-Beijing	Test stat.	*P* value
Total		46	59		
Gender	Female	16 (43.2)	21 (56.8)	Chisq. (1 df) = 0	1.000
	Male	30 (44.1)	38 (55.9)		
Age (year-old)	15~	21 (51.2)	20 (48.8)	Chisq. (2 df) = 1.92	0.383
	35~	12 (35.3)	22 (64.7)		
	60~	13 (43.3)	17 (56.7)		
Ethnicity	Han	43 (47.3)	48 (52.7)	Fisher's exact test	0.365
	Buyi	0 (0.0)	2 (100.0)		
	Miao	1 (25.0)	3 (75.0)		
	Other	2 (25.0)	6 (75.0)		
Occupation	Clerk	3 (42.9)	4 (57.1)	Fisher's exact test	0.953
	Student	3 (37.5)	5 (62.5)		
	Peasant	9 (40.9)	13 (59.1)		
	Migrant labor	31 (45.6)	37 (54.4)		
Education	Primary and below	13 (40.6)	19 (59.4)	Chisq. (2 df) = 0.24	0.888
	Middle school	24 (44.4)	30 (55.6)		
	University and above	9 (47.4)	10 (52.6)		
Marital Status	Single	14 (50.0)	14 (50.0)	Chisq. (2 df) = 0.59	0.743
	Normal marriage status	27 (41.5)	38 (58.5)		
	Abnormal marriage status	5 (41.7)	7 (58.3)		
Monthly income (CNY)	0~	18 (45.0)	22 (55.0)	Fisher's exact test	0.693
	1000~	14 (42.4)	19 (57.6)		
	3000~	10 (38.5)	16 (61.5)		
	5000~	4 (66.7)	2 (33.3)		
Smoke	No	28 (50.0)	28 (50.0)	Chisq. (1 df) = 1.37	0.242
	Yes	18 (36.7)	31 (63.3)		
Alcohol drinking	No	40 (43.5)	52 (56.5)	Chisq. (1 df) = 0	1.000
	Yes	6 (46.2)	7 (53.8)		
Know salt-intake limit	No	28 (37.8)	46 (62.2)	Chisq. (1 df) = 2.86	0.091
	Yes	18 (58.1)	13 (41.9)		
Know oil-intake limit	No	34 (42.0)	47 (58.0)	Chisq. (1 df) = 0.21	0.644
	Yes	12 (50.0)	12 (50.0)		
Regularly serve of fruit-vegetable	No	6 (31.6)	13 (68.4)	Chisq. (1 df) = 0.87	0.351
	Yes	40 (46.5)	46 (53.5)		
Regularly serve of meat	No	5 (26.3)	14 (73.7)	Chisq. (1 df) = 2.08	0.149
	Yes	41 (47.7)	45 (52.3)		
Regular physical exercise	No	43 (42.6)	58 (57.4)	Fisher's exact test	0.317
	Yes	3 (75.0)	1 (25.0)		
Depression	No	41 (46.1)	48 (53.9)	Chisq. (1 df) = 0.68	0.409
	Yes	5 (31.2)	11 (68.8)		
Staying up late	No	41 (44.1)	52 (55.9)	Chisq. (1 df) = 0	1.000
	Yes	5 (41.7)	7 (58.3)		

Family history of DM	No	38 (43.7)	49 (56.3)	Chisq. (1 df) = 0	1.000
	Yes	8 (44.4)	10 (55.6)		
Family history of HTN	No	40 (43.0)	53 (57.0)	Chisq. (1 df) = 0.02	0.881
	Yes	6 (50.0)	6 (50.0)		
Other NCDs diagnosed except for DM and HTN	No	44 (44.9)	54 (55.1)	Fisher's exact test	0.463
	Yes	2 (28.6)	5 (71.4)		
Family history of DM	No	42 (45.2)	51 (54.8)	Chisq. (1 df) = 0.22	0.640
	Yes	4 (33.3)	8 (66.7)		
Family history of HTN	No	35 (44.9)	43 (55.1)	Chisq. (1 df) = 0.02	0.882
	Yes	11 (40.7)	16 (59.3)		
Family history of other NCDs except for DM and HTN	No	31 (43.7)	40 (56.3)	Chisq. (1 df) = 0	1.000
	Yes	15 (44.1)	19 (55.9)		
Family history of TB	No	43 (44.8)	53 (55.2)	Fisher's exact test	0.728
	Yes	3 (33.3)	6 (66.7)		
X-ray	Normal	7 (41.2)	10 (58.8)	Chisq. (1 df) = 0	1.000
	Abnormal	39 (44.3)	49 (55.7)		
Presence of cave	No	34 (45.9)	40 (54.1)	Chisq. (1 df) = 0.22	0.641
	Yes	12 (38.7)	19 (61.3)		
Drug-resistant	No	40 (44.0)	51 (56.0)	Chisq. (1 df) = 0	1.000
	Yes	6 (42.9)	8 (57.1)		
Know TB curable	No	1 (16.7)	5 (83.3)	Fisher's exact test	0.227
	Yes	45 (45.5)	54 (54.5)		
Know air transmission	No	1 (12.5)	7 (87.5)	Fisher's exact test	0.076
	Yes	45 (46.4)	52 (53.6)		
Know PTB symptoms	No	14 (41.2)	20 (58.8)	Chisq. (1 df) = 0.03	0.868
	Yes	32 (45.1)	39 (54.9)		
Know designated hospital	No	2 (22.2)	7 (77.8)	Fisher's exact test	0.293
	Yes	44 (45.8)	52 (54.2)		
Know TB free to be treated	No	8 (30.8)	18 (69.2)	Chisq. (1 df) = 1.73	0.188
	Yes	38 (48.1)	41 (51.9)		
Know TB treat principles	No	11 (35.5)	20 (64.5)	Chisq. (1 df) = 0.81	0.370
	Yes	35 (47.3)	39 (52.7)		
Know NCD non-infectious	No	28 (38.9)	44 (61.1)	Chisq. (1 df) = 1.66	0.197
	Yes	18 (54.5)	15 (45.5)		
Know NCDs & TB sharing co-risk factor	No	36 (43.4)	47 (56.6)	Chisq. (1 df) = 0	1.000
	Yes	10 (45.5)	12 (54.5)		

* *TB: tuberculosis. DM: diabetes mellitus. HTN: hypertension. NCDs: non-communicable chronic diseases, refers to DM, HTN, dyslipidemia, heart disease and COPD. Other NCDs: dyslipidemia, heart disease and COPD. Regularly serve meat/vegetable/fruits: Here relates to serving meat at least one meal per day for three days or greater per week. Same frequency interpretation to Regular physical exercise. Salt-intake limit refers to intake edible salt over 6 grams/day/adult according to the Dietary Guidelines for Chinese Residents (2016). Oil intake limit: Over 30 grams/day/adult according to the Dietary Guidelines for Chinese Residents (2016). Smoke refers to smoking in the past 12 months, including both daily and non-daily smoking. Alcohol drinking refers to drinking in the past 12 months, including both daily and non-daily drinking. CNY: Chinese Yuan.*
